# First record in the Tropical Eastern Pacific of the exotic species *Ficopomatus uschakovi* (Polychaeta, Serpulidae)

**DOI:** 10.3897/zookeys.238.3970

**Published:** 2012-11-05

**Authors:** Rolando Bastida-Zavala, Socorro García-Madrigal

**Affiliations:** 1 Laboratorio de Sistemática de Invertebrados Marinos (LABSIM), Universidad del Mar, campus Puerto Ángel, Ciudad Universitaria, Apdo. Postal 47, Puerto Ángel, Oaxaca, 70902, México

**Keywords:** Annelida, brackish-water, Chiapas, fouling, ecologic impacts, La Encrucijada, México, polychaete taxo-nomy, systematics

## Abstract

The exotic Indo-West-Pacific species, *Ficopomatus uschakovi* (Polychaeta, Serpulidae) is recorded for the first time in the Tropical Eastern Pacific from two sites in La Encrucijada Biosphere Reserve, Chiapas, a coastal lagoon in the Pacific south of Mexico. The means of dispersal of this serpulid species still remains unclear, as the nearest port (Puerto Chiapas) is 70 km to the south, and there are no port installations or shrimp cultures in the lagoon. The record of this serpulid species, apparently widely distributed in this coastal lagoon, has implications regarding possible effects on the brackish-water ecosystem, since the invasion event very well may have occurred several years ago. It is recommended that an exhaustive study be carried out in the coastal lagoons of Chiapas to evaluate the real distribution and the effects of this invasive species on the ecosystem. A complete description, including photographs and drawings, is provided.

## Abstract (en)

Se registra por primera vez la especie exótica del Indo-Pacífico occidental, *Ficopomatus uschakovi* (Polychaeta, Serpulidae), en el Pacífico oriental tropical, en dos sitios de la reserva de la biosfera La Encrucijada, Chiapas, una laguna costera en el Pacífico sur de México. El medio de dispersión de este serpúlido no es claro, debido a que el puerto más cercano (Puerto Chiapas) está 70 km al sur de la laguna, tampoco hay instalaciones portuarias ni estanques de cultivo de camarón. El registro de esta especie de serpúlido, aparentemente bien distribuida en esta laguna costera, tiene implicaciones con respecto a posibles efectos sobre el ecosistema salobre, sobretodo considerando que el evento de invasión pudo suceder hace ya varios años. Se recomienda realizar un estudio exhaustivo en las lagunas costeras de Chiapas para evaluar la distribución real y los efectos de esta especie invasora en el ecosistema. Se incluye una descripción completa, con fotografías e ilustraciones de la especie.

## Introduction

The genus *Ficopomatus* Southern, 1921 includes five species of brackish-water serpulids (ten Hove and Kupriyanova 2009): *Ficopomatus macrodon* Southern, 1921, from India and Sri Lanka (ten Hove and Weerdenburg 1978); *Ficopomatus enigmaticus* (Fauvel, 1923), recorded from subtropical-temperate waters worldwide (Fauvel 1923, ten Hove and Weerdenburg 1978); *Ficopomatus miamiensis* (Treadwell, 1934), widely distributed in the Gulf of Mexico and Caribbean (Rioja 1945, ten Hove and Weerdenburg 1978, Bastida-Zavala and Salazar-Vallejo 2000, and see below); *Ficopomatus uschakovi* (Pillai, 1960), from Indo-Pacific and West Africa (ten Hove and Weerdenburg 1978); and *Ficopomatus talehsapensis* Pillai, 2008, known only from Taléh Sap, Gulf of Thailand (Pillai 2008). It should be noted that we do not follow Pillai (2008), who reinstated the genus *Neopomatus* for the species *uschakovi* on the single autapomorphy of left and right thoracic membranes joined over the thorax.

On the Pacific Coast of America, no indigenous species of *Ficopomatus* has been described (Bastida-Zavala 2008). However, *Ficopomatus enigmaticus*, was recorded as an exotic species from San Francisco Bay, California (ten Hove and Weerdenburg 1978, Bastida-Zavala 2008), and, recently, *Ficopomatus miamiensis* was recorded from Urías lagoon, near Mazatlán, southeast of the Gulf of California, as an exotic species related to shrimp-ponds; however, the introduction could have taken place 20 years ago, when shrimp larvae were imported from Florida (Salgado-Barragán et al. 2004, Tovar-Hernández et al. 2009).

The case of *Ficopomatus enigmaticus*, a serpulid widely dispersed around the world and with a long historical record of invasions, is a well-known example, because it can have dramatic impacts in invaded habitats building large, reef-like colonies in some coastal lagoons that cause major changes in benthic communities, especially in the Lake of Tunis (ten Hove and van den Hurk 1993) and the Mar Chiquita Lagoon, Argentina (Schwindt et al. 2001, Luppi and Bas 2002, Bazterrica et al. 2012). Rioja (1943) recorded, for the first time, *Ficopomatus enigmaticus* from Puerto Quequén, south of Mar del Plata, and now the species is widely distributed in the Mar Chiquita Lagoon, forming annular reefs and having several effects on the ecosystem and navigation of fishing boats in this lagoon-system (Schwindt et al. 2001, Luppi and Bas 2002, Schwindt et al. 2004a, b). Borthagaray et al. (2006) analysed the potential impacts of this invasive species in other lagoons of La Plata River, especially of the Uruguay coast.

The effects of *Ficopomatus miamiensis* in Urías lagoon were considered to be positive in the shrimp-ponds because the population of these serpulids (densities higher than 230,000 ind/m^2^) helps to clean the water and control the suspended particulates. However, the impact of the colonies attached to the mangrove roots is negative, because the serpulid competes with other fouling and filter-feeding invertebrates such as native barnacles, mussels and oysters (Tovar-Hernández et al. 2009).

*Ficopomatus uschakovi* has been recorded from the Gulf of Guinea, West Africa since the 1950’s (Hartmann-Schröder 1971). Recently, this species was recorded in north-eastern Brazil (de Assis et al. 2008) and Venezuela (Liñero-Arana and Díaz-Díaz 2012).

## Materials and methods

From 19–24 September, 2011, we visited several sites to collect marine invertebrates in Chiapas, including Puerto Chiapas (or Puerto Madero, as its old name), La Encrucijada Biosphere Reserve, Chocohuital, Boca del Cielo Lagoon, Paredón (Mar Muerto Lagoon) and ending in Oaxaca, in San Dionisio del Mar (Superior Lagoon). Serpulid samples were taken from hard substrates such as rocks, mangrove roots and artificial structures (e.g. piers, submerged buildings, etc.). The preliminary examination of the specimens included only the samples from La Encrucijada Biosphere Reserve because *Ficopomatus* tubes were immediately identified in the field. In the other sites in Chiapas and Oaxaca the typical tubes of *Ficopomatus* were not found. Most specimen lots were deposited in the Colección de Invertebrados Marinos, Universidad del Mar, Puerto Ángel, Oaxaca (UMAR-Poly), other samples were deposited in the collections of El Colegio de la Frontera Sur, Chetumal, Quintana Roo (ECOSUR), and in the Universidad Autónoma de Nuevo León, Monterrey, México (UANL). Topotypical specimens examined came from the Los Angeles County Museum of Natural History, Allan Hancock Foundation, Los Angeles, California, USA (LACM-AHF).

The specimens were fixed with 10% formalin and preserved in 70% alcohol. The line drawings were made using camera lucida, the photographs were taken with a digital camera (Nikon Coolpix). Opercula were generally found to be covered in silt and algae, and were, therefore, cleaned with a fine brush.

Standard measurements and counts were total length, measured from the most distal part of the operculum to the pygidium; thoracic length, from the apron to the base of the collar; thoracic width, measured across the collar region; the number of thoracic chaetigers, the number of radioles in each lobe of the branchial crown; opercular length, from the base of peduncle to the end plate; and opercular diameter, measured across the dorso-ventral axis of the end plate.

The abbreviations used in the text were as follows: OL (opercular length), OD (opercular diameter), THL (thoracic length), THW (thoracic width), TL (total length of the body), n (sample size), r (range of data), µ (mean), and ± (standard deviation).

## Systematics

### Class Polychaeta Grube, 1850. Family Serpulidae Rafinesque, 1815. Genus *Ficopomatus* Southern, 1921

**Type species.** *Ficopomatus macrodon* Southern, 1921 by monotypy.

#### Ficopomatus uschakovi

Pillai, 1960

http://species-id.net/wiki/Ficopomatus_uschakovi

Figs 1A–E , 2A–I

**Figure 1.**
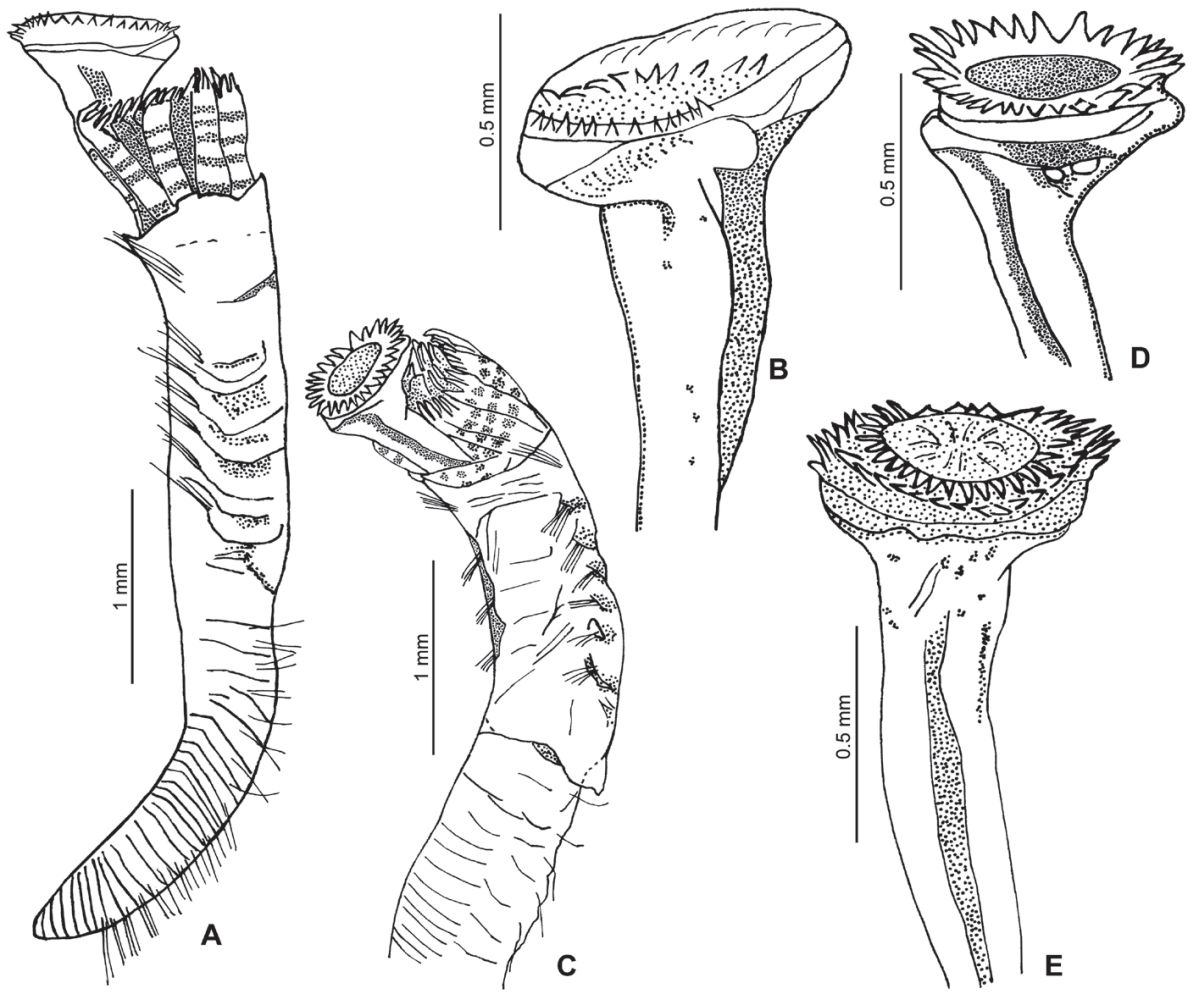
**A–E** *Ficopomatus uschakovi*, from La Encrucijada Biosphere Reserve, UMAR-Poly 112–113. **A, C** complete body in lateral and dorsal views; **B, D–E** opercula, lateral views.

**Figure 2.**
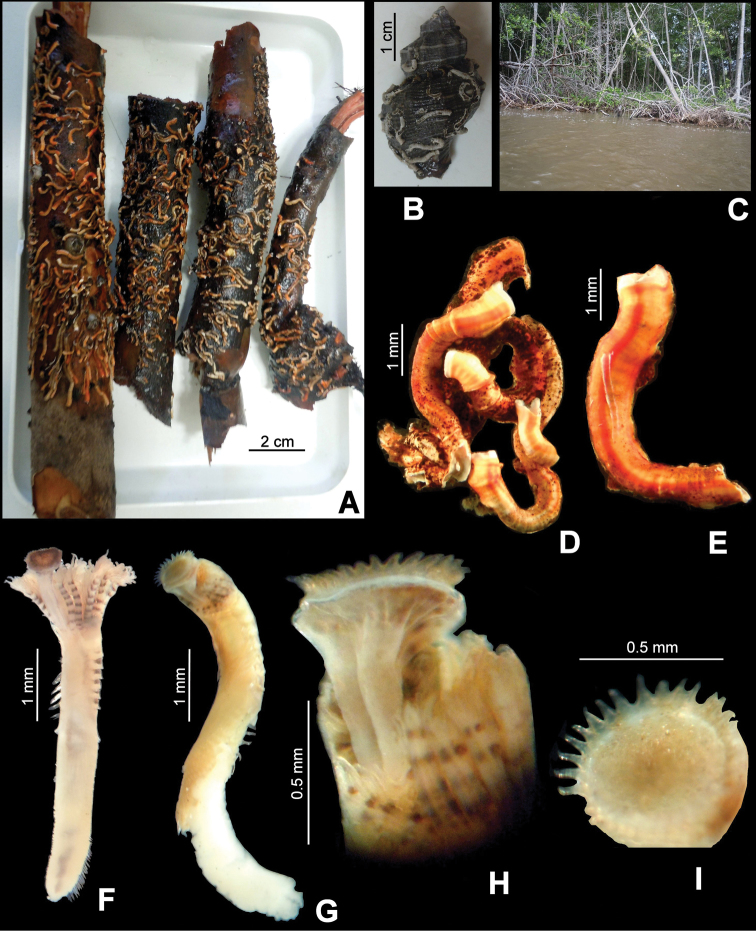
**A–I** *Ficopomatus uschakovi*, from La Encrucijada Biosphere Reserve, UMAR-Poly 112–113. **A** tubes on mangrove roots **B** tubes on the shell of the gastropod *Thaisella kiosquiformis* **C** mangroves in the collecting site **D** tubes forming small aggregations **E** large, single tube **F** complete specimen in dorsal view **G** complete specimen with mass of sperm attached to the abdomen **H** operculum in dorsal view **I** operculum in aboral view.

Neopomatus uschakovi Pillai, 1960: 28–32, text-figs 10H, 11A–H, 12A–H, plate I, figs 1–2; Pillai 2008: 43–49, Fig 5–6, reinstated the genus *Neopomatus*.
Neopomatus uschakovi var. *lingayanensis* Pillai, 1965: 170–172, Fig. 23A–I. Type locality: Lingayan Gulf, Luzon Island, Philippines.
Neopomatus similis Pillai, 1960: 32–33, text-figs 12I–M, plate II, fig. 1. Type locality: Negombo Lagoon, Sri Lanka.
Neopomatus similis var. *rugosus* Pillai, 1960: 33–35, plate II, fig. 2. Type locality: Negombo Lagoon, Sri Lanka.
Mercierella enigmatica (not Fauvel, 1923): Several examples of incorrect use of this name have been studied by ten Hove and Weerdenburg 1978: 109–110.
Ficopomatus uschakovi : ten Hove and Weerdenburg 1978: 109–112, figs 2a–d, 3a, f–k, 4j–n, r, x–z, jj–mm, yy, 5d, revision; de Assis et al. 2008: 51–58, Fig. 2A–G, Brazil; Liñero-Arana and Díaz-Díaz 2012: 234–237, fig. 1a–j, Venezuela.

##### Type locality.

Panadura River estuary, Madu Ganga estuary at Balapitiya and Ratgama Lake at Dodanduwa, Sri Lanka.

##### Material examined. 

**Chiapas, South Pacific of Mexico.** Five specimens (ECOSUR), three specimens (UANL), more than 100 specimens (UMAR-Poly 112), La Encrucijada Biosphere Reserve, Barra San Juan, 15°09'58"N, 92°51'12"W, 0.5–1 m, submerged mangrove root (*Rhizophora mangle*), September 21, 2011, Rolando Bastida-Zavala et al. leg. Five specimens (ECOSUR), 20 specimens (UMAR-Poly 113), La Encrucijada Biosphere Reserve, Zacapulco, 15°11'37"N, 92°53'22"W, on rotting mangrove root and gastropod shell, 0–0.5 m, September 21, 2011, Rolando Bastida-Zavala et al. leg.

##### Topotypical material.

Two specimens (LACM-AHF N10947), Panadura River estuary, Sri Lanka, brackish water (donated by T.G. Pillai), October 9, 1961.

##### Description.

Mass occurrence is present (Fig. 2A, D); however, some specimens were solitary (Fig. 2E). The tube colour varies from pink to red in live material, changing to white, brown or orange in preserved material (Fig. 2D–E). They possess several prominent to shallow peristomes (Fig. 2D) or only low growth rings (annulations, Fig. 2E), but lack longitudinal ridges and alveoli.

The body is yellow; with 6–7 dark bands on the radioles (preserved material, Figs 1A, 2F). The thorax has five dark bands on the lateral side, between the notopodial bundles, although sometimes these bands are blurred. TL=6.7 mm (n=10, r:4–6.7, µ=5.2 ±1); THL=2 mm (n=10, r:1.4–2, µ=1.7 ±0.2); THW=0.7 mm (n=10, r:0.5–0.7, µ=0.6 ±0.1). The branchial crown has nine radioles (n=10, r:6–9, µ=7.4 ±0.8) on the left, and eight on the right (n=10, r:7–9, µ=8 ±0.7). Interradiolar membrane absent.

The peduncle is smooth, inserted in the left branchial lobe; lacks a constriction (Fig. 1D–E); OL=1.8 mm (n=9, r:1–1.9, µ=1.6 ±0.4). The operculum is spherical to oval in shape, with a slightly convex, flat or slightly concave horny plate (Fig. 1B–E). OD=0.6 mm (n=9, r:0.5–0.8, µ=0.6 ±0.1). The end plate bears 1–5 concentric rows of spines (Fig. 2H); the rows are sometimes incomplete or converge with the other rows. The spines are transparent (Fig. 2I).

The collar is entire, with well-developed lobes. The collar chaetae include coarsely serrated chaetae and hooded (capillary) chaetae. The thoracic membranes are fused dorsally, ventrally forming a small apron. The thorax has six chaetigers with hooded (limbate) chaetae, and saw-shaped uncini. A short achaetous region is present between the thorax and abdomen. Most of the abdominal segments possess geniculate chaetae and rasp-shaped uncini.

##### Distribution.

**Figure 3.**
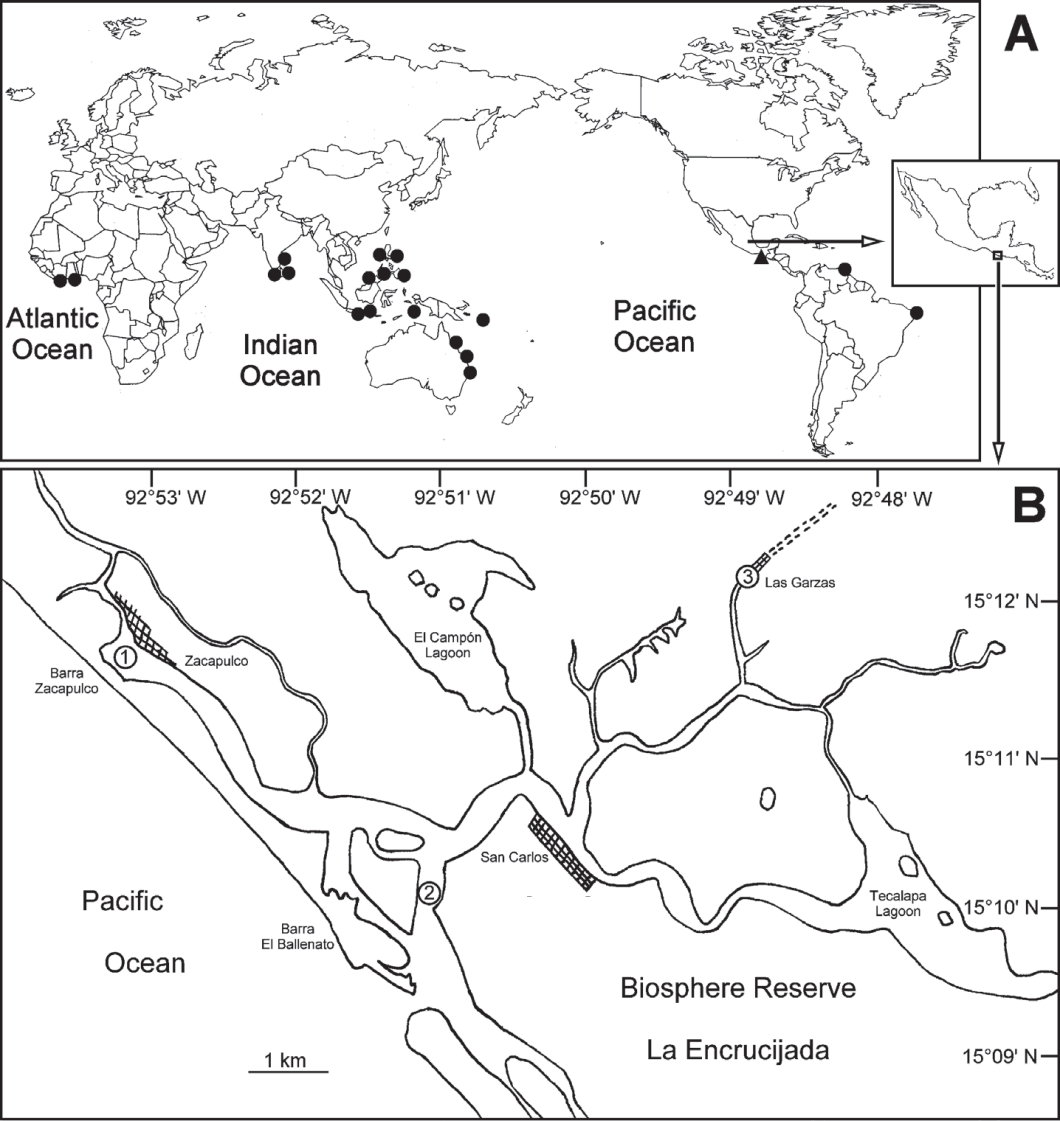
**A** World-wide distribution of *Ficopomatus uschakovi*. Triangles denote examined material, circles literature records (data from ten Hove and Weerdenburg 1978) **B** Study area and the localities where specimens of *Ficopomatus uschakovi* were recollected. **1**: Zacapulco; **2**: Barra San Juan; **3**: Las Garzas boat pier (observed by S.I. Salazar-Vallejo et al., pers. comm.).

Originally limited to Indo-West Pacific and Gulf of Guinea (ten Hove and Weerdenburg 1978), the species was also recorded in some isolated areas of the tropical and subtropical Western Atlantic: Sossego Creek, Brazil (de Assis et al. 2008), Morocoto Creek, Venezuela (Liñero-Arana and Díaz-Díaz 2012) (Fig. 3A) and several sites in the Northern Gulf of Mexico (Bastida-Zavala et al. in prep.). There have been new records in Chiapas coast, Southern Pacific of Mexico (Fig. 3B).

##### Ecology.

Depth: Intertidal to 1 m. On mangrove roots (Fig. 2A, C) and on the shell of the gastropod *Thaisella kiosquiformis* (Duclos, 1832) (Fig. 2B); coastal lagoon with salinity ranges approximately 20–35 o/oo. On shells, stones and petiole bases of coconut leaves in the topotypical area (Pillai 1960); on submerged piece of bamboo, with salinity ranges approximately 4–11 o/oo in Brazil records (de Assis et al. 2008).

##### Reproductive characters.

Two specimens (TL=4 mm and 4.2 mm) have masses of sperm glued to the abdomen (Fig. 2G); however, eggs have not been found in the specimens studied in detail (n=10).

##### Remarks.

The specimens of *Ficopomatus uschakovi* from Chiapas are slightly bigger than the topotypical specimens (TL=4–4.2 mm, THL=1.1–1.4 mm, THW=0.7–0.8 mm, OL=1.1–1.2, OD=0.3–0.5 mm); however, the rest of the morphological characteristics are very similar.

Apart from the spines of the operculum, the main character that separates *Ficopomatus uschakovi* from *Ficopomatus enigmaticus* and *Ficopomatus miamiensis* is the dorsally fused thoracic membranes. This autapomorphy of *Ficopomatus uschakovi* is regarded to be of generic level by Pillai (2008), but is not followed by us.

In the local community the serpulid tubes on the mangroves are called ‘broca’ (more or less ‘drill’), just like all other sessile invertebrates with calcareous covers, such as barnacles and oysters.

## Discussion

The presence of a species, resembling *Ficopomatus uschakovi*, from La Encrucijada Biosphere Reserve was initially brought to the attention of the authors two years ago (S.I. Salazar-Vallejo et al. pers. comm. 2009). Previously, no other record of the species existed throughout the Tropical Eastern Pacific. However, the observations in the field show that *Ficopomatus uschakovi* is widely distributed in La Encrucijada, suggesting that the invasion event of this species may have occurred several years ago.

Although ballast water, sediment transport and fouling are the main means of dispersal of aquatic exotic species (Carlton and Geller 1993, Ruiz et al. 2000, Okolodkov et al. 2007), the means of dispersal of *Ficopomatus uschakovi* in La Encrucijada Biosphere Reserve still remains unclear, as the nearest port (Puerto Chiapas) is 70 km to the south, and there are no port installations or any shrimp cultures in the lagoon. The local people mentioned that these serpulids had gone unnoticed until now and apparently negative effects to the ecosystem have not been detected, except the fouling of the fishing boats, which require frequent cleaning.

Mass occurrence of *Ficopomatus uschakovi* was observed (Fig. 2A), however, not to the extent of the reef-like structures, formed by *Ficopomatus enigmaticus* in Argentina (Schwindt et al. 2004a, b) and by *Ficopomatus miamiensis* in Mazatlán (Tovar-Hernández et al. 2009). However, similar impacts as caused by the latter species may be expected for *Ficopomatus uschakovi*. Therefore, it is necessary to conduct detailed monitoring of *Ficopomatus uschakovi* in the sites that have been invaded. It is also recommend that an exhaustive study in the coastal lagoons of Chiapas be conducted to evaluate the real distribution and the impact generated by *Ficopomatus uschakovi*.

## Supplementary Material

XML Treatment for Ficopomatus uschakovi
